# Complete Remission of Advanced Hepatocellular Carcinoma in a Patient With Ulcerative Colitis Treated With Atezolizumab and Bevacizumab: A Case Report

**DOI:** 10.7759/cureus.39538

**Published:** 2023-05-26

**Authors:** Swetha Pentapati, Stephen Caucci, Shravya Balmuri, Vishal Devarkonda

**Affiliations:** 1 Hematology and Oncology, Michigan State University College of Human Medicine, Lansing, USA; 2 Internal Medicine, Michigan State University College of Human Medicine, Lansing, USA; 3 Internal Medicine, Louisiana State University Health Sciences Center, Shreveport, USA

**Keywords:** chronic alcoholism, alcoholism, atezolizumab and bevacizumab, ulcerative colıtıs, ulcerative, hepatocellular carcinoma (hcc)

## Abstract

In this case study, a 73-year-old man who had previously undergone colectomy had a history of ulcerative colitis and alcohol abuse and presented with fatigue, weight loss, and a liver lesion. After a biopsy, he was diagnosed with stage IV-A hepatocellular carcinoma with poor differentiation and cirrhotic architecture, and molecular testing revealed positivity for multiple genes. A combination of atezolizumab and bevacizumab was administered, resulting in complete remission lasting beyond 16 months, demonstrating the potential of these drugs as a treatment option for advanced hepatocellular carcinoma (HCC). The patient's history of autoimmune conditions could have contributed to his robust response to the treatment. The report highlights the sustained survival benefits of this treatment beyond month 16.

## Introduction

The most common primary malignancy of the liver is hepatocellular carcinoma (HCC), which ranks as the fourth leading cause of cancer-related death. Treatment for HCC primarily relies on the Barcelona Clinic Liver Cancer treatment strategy, which considers tumor, node, metastasis (TNM) staging, performance status using an Eastern Cooperative Oncology Group (ECOG) score, and liver functionality with a Child-Pugh score [1]. Patients with early-stage disease, good performance status, and liver functionality can undergo resection or transplantation. However, minimal options exist for more advanced stages of HCC, with kinase inhibitors such as Sorafenib and Lenvatinib being approved as first-line therapy for non-resectable HCC [2]. Although they have shown a survival benefit, their side effects can negatively impact patients' quality of life. Recently, the IMbrave150 trial demonstrated better overall and progression-free survival with atezolizumab and bevacizumab compared to sorafenib, with better tolerability [1]. However, it is unclear how long the survival benefit lasts beyond month 16 and if complete remission is achievable and sustainable. In this case report, we discuss a patient who achieved a robust response to atezolizumab and bevacizumab, leading to complete remission and has remained in remission for one year without active treatment.

## Case presentation

A 73-year-old male with a past medical history of ulcerative colitis, a colectomy in 2009, hyperlipidemia, obstructive sleep apnea, hypothyroidism, and paroxysmal atrial fibrillation presented to the emergency department on 4/31/2021. He complained of increasing malaise and generalized weakness with fatigue, decreased appetite, epigastric pain, and unintentional weight loss of 10-15 pounds over a month. The patient had a history of smoking (15 packs per year) and current alcohol use of 10 beers daily for 30 years. His family history includes his father having prostate cancer and his brother having bladder cancer.

Physical examination

The patient’s physical examination was unremarkable except for sinus tachycardia.

Lab findings

The patient's complete blood count (CBC) and comprehensive metabolic panel (CMP) were unremarkable. Serology studies revealed an Alpha-fetoprotein of 9495 U/L, CA19-9 of 147.5 U/mL, and carcinoembryonic antigen (CEA) of 2 ng/mL. HIV and Hepatitis C screening was negative.

Imaging

**Figure 1:**
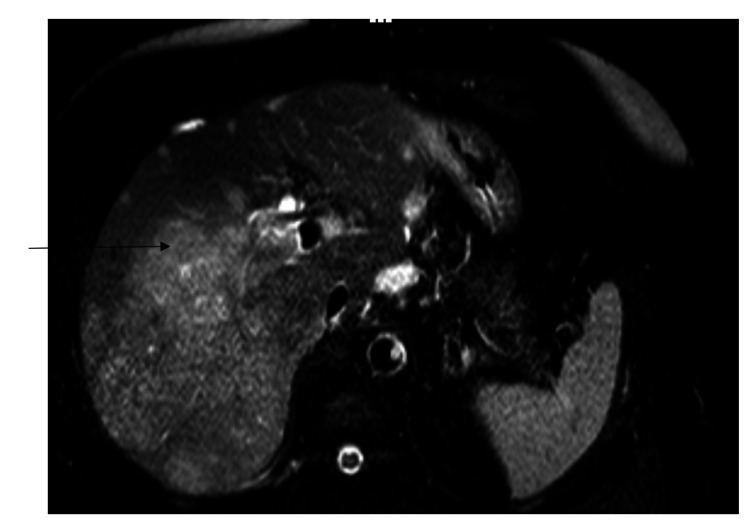
MRI abdomen with and without contrast with an arrow pointing to a lobulated heterogeneously enhanced infiltrative mass involving nearly the entire right lobe of the liver

A CT scan of the chest with contrast was performed, which revealed no evidence of pulmonary embolism but demonstrated a 2.4 cm enhancing lesion on the right lobe of the liver with right pericardial lymphadenopathy. Further workup with MRI abdomen (Figure 1) with and without contrast showed a lobulated heterogeneously enhancing infiltrative mass involving nearly the entire right lobe of the liver, scattered subcentimeter lesions in the left liver lobe, a lobulated heterogeneous mass in the porta hepatis, and evidence of thrombosis of the right portal vein. 

Biopsy and diagnosis

The patient underwent a biopsy of the right hepatic lobe mass, revealing an HCC poorly differentiated with cirrhotic architecture. The tumor was faint to moderately positive for alpha-fetoprotein and human serum albumin (HSA), and further staining for lymphocyte common antigen (CD45), neural cell adhesion molecule (CD56), tumor protein 63 (P63), synaptophysin, thyroid transcription factor 1 (TTF1), iron storage, and trichrome stain supported cirrhosis architecture. Molecular testing was limited due to a small tissue sample, but genetics were positive for tumor protein P53 (TP53), nuclear factor erythroid 2-related factor 2 (NFE2L2), catenin beta-1 (CTNNB1), the telomerase reverse transcriptase (TERT) promoter, and AT-rich interactive domain-containing protein 1A (ARID1A). Biomarkers were positive for MutL homolog 1 (MLH1), MutS homolog 2 (MSH2), MutS homolog 5 (MSH5), and postmeiotic segregation increased 2 (PMS2) but negative for programmed death-ligand 1 (PD-L1).

Clinical course with follow-up imaging

**Figure 2:**
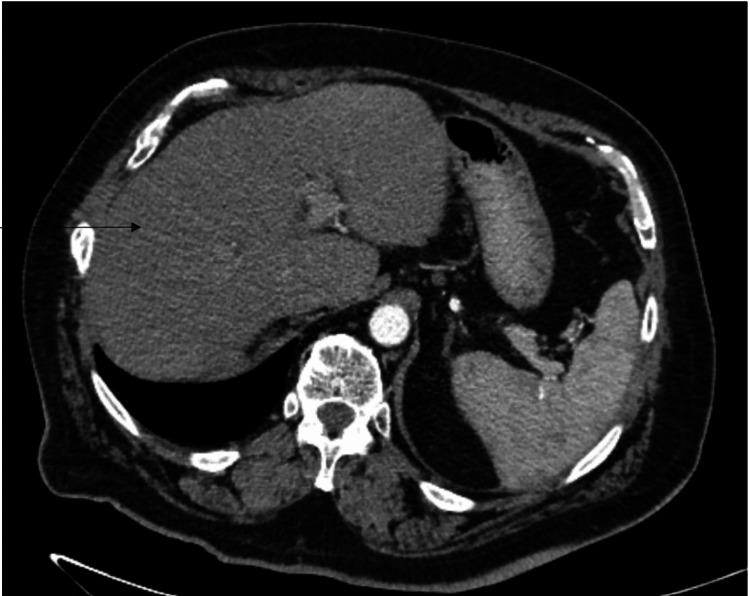
Stable liver appearance and absence of metastatic disease in chest, abdomen, and pelvis: follow-up CT imaging one year after treatment: arrow indicates no evidence of residual or recurrent disease

The patient was determined to have stage IV-A hepatocellular carcinoma (pT4 cN1 cM0) and was started on Atezolezumab 1200 mg and Bevacizumab 15 mg/kg on 5/20/2021 every three weeks. Follow-up lab work since treatment showed undetectable alpha-fetoprotein (AFP) in August 2021. In winter 2021, the patient relocated to Arizona and received bevacizumab and atezolizumab every three weeks. A follow-up MRI in October 2021 showed a marked decrease in tumor burden and lymphadenopathy, with a re-demonstration of portal vein thrombosis. In February 2022, the patient got into an accident and sustained multiple injuries. The patient decided to stop receiving immunotherapy in February 2022. The patient underwent cervical spine surgery in May 2022. In June 2022, CT chest/abdomen/pelvis showed atrophy of the right hepatic lobe posterior segment without convincing evidence for local recurrence, porta hepatis and right epicardial lymphadenopathy unchanged from 10/27/21, no evidence of new metastases, and unchanged lymphadenopathy. On follow-up with oncology, the patient restarted treatment with bevacizumab and atezolizumab on 6/23/22. However, following treatment, the patient experienced confusion, dehydration, fatigue, and falls, requiring an ER visit for fluid resuscitation. After a discussion with the patient and his family in July 2022, a shared decision was made to hold further treatment and monitor him while he works with physical therapy to improve his performance. In October 2022, the patient followed up with his oncologist for a repeat CT chest/abdomen/pelvis (Figure 2), demonstrating a stable appearance of the liver with no evidence of residual or recurrent disease, no evidence of metastatic disease in the chest, abdomen, and pelvis, stable borderline enlarged lymph nodes in the periportal region, and no new, enlarging, or suspicious development of pulmonary nodules. AFP also remains undetectable. At this time, the patient chose to be monitored out of treatment.

Final diagnosis

Stage 4 hepatocellular carcinoma with a robust response to atezolizumab and bevacizumab and complete remission at 16 months.

## Discussion

Hepatocellular carcinoma (HCC) is a rapidly growing tumor commonly affecting patients with cirrhosis and hepatitis B viral infection, with over half a million new cases diagnosed annually worldwide. Early detection, surveillance, and effective treatment are crucial for improving survival in patients with primary liver malignancies, which have a low five-year survival rate and rank as the fourth most common cause of cancer-related death worldwide. Treatment options for HCC depend on the extent of the disease and may include surgical and non-surgical modalities. The Barcelona Clinic Liver Cancer (BCLC) algorithm stages patients and guides treatment decisions [3].

This case study details a patient with stage IV-A hepatocellular carcinoma with portal vein thrombosis who received a combination of atezolizumab and bevacizumab systemic chemotherapy since liver transplantation was not feasible due to macrovascular tumor invasion. After undergoing eight treatment cycles, the patient showed a complete response with undetectable AFP, stable disease, and no disease progression on CT imaging. The case adds to other reports of successful responses to atezolizumab and bevacizumab [4,5]. This case report highlights whether treatment can be stopped after achieving a complete response in hepatocellular carcinoma (HCC) or if surveillance alone is sufficient. Two cases with similar outcomes using a combination of atezolizumab and bevacizumab are discussed. In one case, the patient achieved complete remission after nine doses of the combination therapy and stopped treatment. In another case, two cycles of combination therapy led to the regression of portal vein thrombosis, enabling surgical resection with sustained remission. These findings suggest the potential for treatment discontinuation and surveillance after achieving complete remission in HCC patients, but further research is needed to determine the optimal management approach [4,5].

According to the Barcelona Clinic Liver Cancer algorithm, tumor invasion leading to portal vein thrombosis leads to a poorer prognosis and limits the therapeutic options available to physicians. The survival rate of patients with advanced disease, including those with portal vein thrombosis, is 10-24 months with supportive care [6]. However, the first-line combination treatments atezolizumab-bevacizumab and durvalumab-tremelimimab have shown more significant overall survival benefits for those with non-resectable advanced-stage HCC compared to the standard of care, sorafenib. Atezolizumab-bevacizumab and durvalumab-tremelimimab have been approved since 2020 and 2022, respectively, increasing the number of approved treatments available [7,8].

Patients with autoimmune diseases have been underrepresented in clinical trials, especially those with checkpoint inhibitors. Despite having a history of ulcerative colitis treated with resection, our patient responded well to atezolizumab-bevacizumab, which has demonstrated safety and efficacy in recent studies [1,6]. However, evidence suggests that patients with better-controlled autoimmune diseases may have better outcomes and fewer autoimmune-related side effects when initiating checkpoint inhibitor treatment. Although no head-to-head trials compare results between autoimmune and non-autoimmune patients, our case suggests that checkpoint inhibitors could be safe and effective in treating autoimmune diseases [9-15]. We hypothesize that patients with hepatocellular carcinoma and autoimmune conditions may respond more favorably to atezolizumab-bevacizumab than patients without autoimmune disorders. Nevertheless, more extensive randomized controlled studies are necessary to understand these conditions better.

## Conclusions

The case report details a patient with advanced stage 4 hepatocellular carcinoma who showed a strong response to treatment with atezolizumab and bevacizumab, resulting in complete remission and no signs of recurrence after 16 months. This suggests that combination therapy may provide a lasting benefit for similar patients. While there is no direct comparison between autoimmune and non-autoimmune patients, this case adds to the growing evidence that checkpoint inhibitors can be safe and effective for treating advanced cancers in individuals with autoimmune diseases.
